# Learner Attitudes on Interprofessional Education Through Formal Interprofessional Case Discussions

**DOI:** 10.1093/geroni/igaa057.704

**Published:** 2020-12-16

**Authors:** Anne Halli-Tierney, Dana Carroll, Robert McKinney, Rebecca Allen

**Affiliations:** 1 The University of Alabama, Tuscaloosa, Alabama, United States; 2 Auburn University Harrison School of Pharmacy, Tuscaloosa, Alabama, United States; 3 Alabama Research Institute on Aging, Tuscaloosa, Alabama, United States

## Abstract

Interprofessional education case sessions allow learners to apply discipline-specific knowledge to real-life scenarios through facilitated discussion of a patient case. Our interprofessional case discussion was implemented for learners to develop care plans for complex geriatric patients; learners have intentional time to learn with, from and about each other’s roles in geriatric patient care. Cases were de-identified from actual complex patients seen in geriatrics clinic. All learners receive the case and work through it from their discipline’s perspective, then join a facilitated group discussion to develop collaborative care plans. At session end participants are surveyed using the ICCAS and qualitative comments about perceptions on interprofessional case learning. Thirty-five learners (47%) completed the feedback survey. Disciplines represented were medicine, pharmacy, psychology and social work. Thirty learners (85%) indicated the case discussion session was very educational (n=22, 62%) to educational (n=8, 23%). Themes used most frequently regarding what was most educational were: “different professional approaches”, “professional roles”, “collaboration” and “problem solving”. Typically, learners were unable to identify “least educational” components to the activity, but some learners found the pace of information presentation too rapid or felt other professionals did not provide enough context for the suggestions they made for the case. Suggestions to improve the interprofessional case discussion activity included increased “time for discussion and consensus building”, more session structure, and addition of other professionals (e.g., nutrition, law). The majority of interprofessional learners participating in interprofessional case sessions have found them to be educational. Feedback from learners aligns with goals of interprofessional education.

